# Loss of KCNJ10 protein expression abolishes endocochlear potential and causes deafness in Pendred syndrome mouse model

**DOI:** 10.1186/1741-7015-2-30

**Published:** 2004-08-20

**Authors:** Philine Wangemann, Erin M Itza, Beatrice Albrecht, Tao Wu, Sairam V Jabba, Rajanikanth J Maganti, Jun Ho Lee, Lorraine A Everett, Susan M Wall, Ines E Royaux, Eric D Green, Daniel C Marcus

**Affiliations:** 1 Anatomy & Physiology Department, Kansas State University, Manhattan, Kansas, USA; 2 Genome Technology Branch, National Human Genome Research Institute, National Institutes of Health, Bethesda, Maryland, USA; 3 Department Medicine, Renal Division, Emory University, School of Medicine, Atlanta, Georgia, USA

## Abstract

**Background:**

Pendred syndrome, a common autosomal-recessive disorder characterized by congenital deafness and goiter, is caused by mutations of SLC26A4, which codes for pendrin. We investigated the relationship between pendrin and deafness using mice that have (*Slc26a4*^+/+^) or lack a complete *Slc26a4 *gene (*Slc26a4*^-/-^).

**Methods:**

Expression of pendrin and other proteins was determined by confocal immunocytochemistry. Expression of mRNA was determined by quantitative RT-PCR. The endocochlear potential and the endolymphatic K^+ ^concentration were measured with double-barreled microelectrodes. Currents generated by the stria marginal cells were recorded with a vibrating probe. Tissue masses were evaluated by morphometric distance measurements and pigmentation was quantified by densitometry.

**Results:**

Pendrin was found in the cochlea in apical membranes of spiral prominence cells and spindle-shaped cells of stria vascularis, in outer sulcus and root cells. Endolymph volume in *Slc26a4*^-/- ^mice was increased and tissue masses in areas normally occupied by type I and II fibrocytes were reduced. *Slc26a4*^-/- ^mice lacked the endocochlear potential, which is generated across the basal cell barrier by the K^+ ^channel KCNJ10 localized in intermediate cells. Stria vascularis was hyperpigmented, suggesting unalleviated free radical damage. The basal cell barrier appeared intact; intermediate cells and KCNJ10 mRNA were present but KCNJ10 protein was absent. Endolymphatic K^+ ^concentrations were normal and membrane proteins necessary for K^+ ^secretion were present, including the K^+ ^channel KCNQ1 and KCNE1, Na^+^/2Cl^-^/K^+ ^cotransporter SLC12A2 and the gap junction GJB2.

**Conclusions:**

These observations demonstrate that pendrin dysfunction leads to a loss of KCNJ10 protein expression and a loss of the endocochlear potential, which may be the direct cause of deafness in Pendred syndrome.

## Background

Pendred syndrome is a relatively common autosomal-recessive disorder characterized by deafness and goiter [1]. The syndrome is caused by mutations of the *PDS *gene SLC26A4, which codes for the protein pendrin [2]. Deafness is congenital and generally profound although sometimes late in onset and provoked by light head injury. Vestibular dysfunction is uncommon. Goiter is variable and generally develops around puberty [3]. The cause of goiter appears to be an impairment of iodide fixation in the follicular lumen due to a reduced rate of iodide transport across the apical membrane of thyroid gland epithelial cells [4]. A positive perchlorate discharge test and an enlarged vestibular aqueduct appear to be the most reliable clinical signs of Pendred syndrome [5].

Pendrin is an anion exchanger that can transport Cl^-^, I^-^, HCO_3 _^- ^and formate [6,7]. Expression has been found in the inner ear and thyroid gland consistent with the clinical signs of deafness and goiter [2,3,8]. In addition, pendrin expression has been found in the kidney [9], mammary gland [10], uterus [11], testes [12] and placenta [13]. No expression was found in fetal or adult brain, consistent with a peripheral cause of deafness [2,11].

Expression of pendrin mRNA in the inner ear has been found in several places including the cochlea, the vestibular labyrinth and the endolymphatic sac [8]. The precise location of pendrin protein expression, however, has not yet been determined. The variability of deafness in Pendred syndrome and the observation that deafness is sometimes late in onset suggest that pendrin dysfunction may not be the direct cause of deafness. It is conceivable that pendrin dysfunction favors changes in the expression levels of proteins that are critical for the maintenance of the hearing function. Detailed studies aimed at identifying the direct cause of deafness in Pendred syndrome have recently become possible due to the generation of a pendrin-specific polyclonal antibody [9] and the development of *Slc26a4*^-/- ^mice, which bear a targeted disruption of the mouse *Slc26a4 *gene [14]. The aim in the present study was to determine the location of pendrin and the cause of deafness in *Slc26a4*^-/- ^mice.

## Methods

The endocochlear potential and the endolymphatic and perilymphatic K^+ ^concentrations were measured in young adult mice (1–4 month of age) that either have (*Slc26a4*^+/+^) or lack (*Slc26a4*^-/-^) a functional gene for pendrin [14]. The mouse strain 129Sv/Ev (Taconic, Germantown, NY) was chosen as the source of *Slc26a4*^+/+ ^controls, since *Slc26a4*^-/- ^mice were propagated in the this strain and generated using a stem cell line derived from 129Sv/Ev. *Slc26a4*^+/+ ^and *Slc26a4*^-/- ^were agouti. They did not differ in coat color. Differences in pigmentation were verified using coisogenic age-matched *Slc26a4*^+/+ ^and *Slc26a4*^-/- ^that were bred in parallel.

Expression of key proteins involved in the generation of the endocochlear potential and the transport of K^+ ^was studied using confocal immunocytochemistry and quantitative RT-PCR. K^+ ^secretion and the generation of the endocochlear potential were measured using electrophysiological techniques. All experiments were approved by the Institutional Animal Care and Use Committee of Kansas State University.

### Confocal immunocytochemistry

Animals were deeply anesthetized with sodium pentobarbital (100 mg/kg i.p.) and transcardially perfused with a Cl^-^-free phosphate-buffered Na-gluconate solution containing 4% paraformaldehyde. Temporal bones were removed and cochleae fixed by perilymphatic perfusion, decalcified in EDTA, processed through a sucrose gradient and infiltrated with polyethylene glycol. Mid-modiolar cryosections (12 μm, CM3050S, Leica, Nussloch, Germany) were blocked in PBS with 0.2% Triton-X (PBS-TX) and 10% bovine serum albumin. Slides were incubated overnight at 4°C with primary antibodies in PBS-TX with 1–3% BSA [rabbit anti-pendrin, 1:500 (h766–780); rat anti-ZO-1, 1:100 (Chemicon, Temecula, CA); goat anti-KCNQ1, 1:200 (C20, Santa Cruz Biotechnology, Santa Cruz, CA), rabbit anti-KCNE1, 1:200 (Alomone, Jerusalem, Israel); rabbit anti-KCNJ10, 1:300 (Alomone); rabbit anti-SLC12A2, 1:100 (Chemicon); and rabbit anti-connexin 26, 1:100 (Zymed, San Francisco, CA)]. Slides were washed in PBS-TX and incubated for 1 h at 25°C with appropriate secondary antibodies at a 1:1000 dilution in PBS-TX with 1–3% BSA [donkey anti-rabbit Alexa 488, chicken anti-rat Alexa 594, and chicken anti-goat Alexa 594 (Molecular Probes, Eugene, OR)]. Actin filaments were visualized by staining with Alexa 488 conjugated phalloidin (Molecular Probes). After incubation, slides were washed with PBS-TX, mounted with FluorSave (Calbiochem, La Jolla, CA), and viewed by confocal microscopy (LSM 5 Pascal or LSM 510 Meta, Carl Zeiss, Jena or Göttingen, Germany). Laser scanning brightfield images were collected to document structural preservation, for morphometric analysis and for analysis of pigmentation.

### Quantitative RT-PCR

Animals were deeply anesthetized with sodium pentobarbital (100 mg/kg i.p.). Kidneys and brain were removed and pulverized in liquid N_2_. Temporal bones were removed and two preparations of inner ear tissues were obtained by microdissection: (1) stria vascularis without spiral ligament, and (2) spiral ganglia including the surrounding bone and the organ of Corti. The dissection medium was changed twice during the microdissection and isolated tissues were washed three times to minimize cross-contamination. The *Slc26a4*^-/-^genotype was verified by the observation of large otoconia in the utricular macula [14]. Total RNA was isolated and residual DNA contamination was removed by enzymatic digestion (RNeasy micro, Qiagen, Valencia, CA). Quality and quantity of 18S rRNA obtained from kidneys and brain were determined (Nano Assay, 2100 Bioanalyzer, Agilent, Palo Alto, CA). Further, the quality of RNA preparations obtained from stria vascularis and spiral ganglia was assessed (Pico Assay, 2100 Bioanalyzer). Real time RT-PCR was performed on RNA obtained from individual animals (OneStep RT-PCR, Qiagen; Smart Cycler, Cepheid, Sunnyvale, CA) in the presence of 0.2× SYBR green I (Molecular Probes). Transcripts of 18S rRNA and mRNA for the K^+ ^channels KCNJ10, KCNQ1 and KCNQ4 were amplified using gene-specific primers (Table 1). RT was performed for 30 min at 50°C and 15 min at 95°C. PCR consisted of 50 cycles of 1 min at 60°C, 1 min at 72°C, 7s heating to hot-measurement temperature, 13s hot-measurement at 3–5°C below product melting temperature (Table 1) and 1 min at 94°C. Hot-measurements were performed to eliminate the detection of primer-dimers that had melting temperatures between 72 and 75°C [15]. PCR was followed by a melt (60 to 95°C). The generation of a single product of appropriate size was routinely checked by the presence of a single melt peak and by agarose gel-electrophoresis. Product identity was confirmed by sequencing.

**Table 1:** Primers

Template	Primers	Product Length	Melting Temperature	Hot Measurement	Fidelity	n
18S	gag gtt cga aga cga tca ga (*sense*)	316 bp	83.2 ± 02°C	78°C	1.89 ± 0.02	17
	tcg ctc cac caa cta aga ac (antisense)					
KCNJ10	tgg tgt ggt gtg gta tct gg (*sense*)	411 bp	83.2 ± 02°C	78°C	1.86 ± 0.02	15
	tga agc agt ttg cct gtc ac (*antisense*)					
KCNQ1	ttt gtt cat ccc cat ctc ag (*sense*)	239 bp	82.5 ± 02°C	79°C	1.85 ± 0.02	17
	gtt gct ggg tag gaa gag (*antisense*)					
KCNQ4	ccc gga aac cct tct gtg tc (*sense*)	245 bp	83.2 ± 02°C	80°C	1.87 ± 0.01	17
	aaa gat gag cac cag gaa cc (*antisense*)					

Template molecules (T) were quantified according to T = P / (F ^C_t_) where P is the number of product molecules, F is the fidelity of the reaction and C_t _is the cycle at which the number of product molecules reaches a chosen threshold [15]. Fidelity (F) was obtained from the slope (S) of the log-linear phase of the growth curve via a best-fit fifth-order polynomial: F = 7.39 + 3.80 × S + 1.05 × S^2 ^+ 0.15 × S^3 ^+ 11.38 × 10^-3 ^* S^4 ^+ 3.39 × 10^-4 ^× S^5^. The number of product molecules at threshold (P_Ct_) was determined by amplification of known amounts of 18S rRNA according to P_Ct _= T × F ^C_t_. Quantifications of 18S rRNA were used to compare tissue amounts. Genomic contamination of inner ear samples was assessed to be <0.02% by omission of the RT step.

### In vitro electrophysiology

Animals were deeply anesthetized with sodium pentobarbital (100 mg/kg i.p.). Stria vascularis without spiral ligament was obtained by microdissection. Currents generated by the stria marginal epithelium were recorded [16]. A Pt-Ir wire microelectrode with a Pt-black tip was positioned 20–30 μm from the apical surface of the epithelium and vibrated at 200–400 Hz by piezo-electric bimorph elements (Applicable Electronics, Forest Dale, MA; ASET version 1.05, Science Wares, East Falmouth, MA). A Pt-black electrode (26-gauge) served as reference in the bath chamber. The signals from the phase-sensitive detectors were digitized (16 bit) at a rate of 0.5 Hz. The output was expressed as current density at the electrode.

### In situ electrophysiology

Animals were anesthetized with inactin (thiobutabarbital sodium salt, 140 mg/kg ip). The endocochlear potential and the endolymphatic [K^+^] were measured with double-barreled microelectrodes [17]. Measurements were made in the basal turn by a round-window approach through the basilar membrane and in the apical turn after thinning the bone over the stria vascularis and picking a small hole (~30 μm). K^+^-selective electrodes were calibrated in solutions of constant cation (K^+ ^and Na^+^) concentration of 150 mM. The minor selectivity of the K^+ ^electrodes for Na^+ ^produced a nonlinearity in the calibration curve, which was closely fitted by the Nicolski equation using nonlinear curve-fitting software (OriginLab, Northampton, MA): V = V_i _+ *S *× log ([K^+^] + *A *× [Na^+^]), where *V*_i _is an offset term, *S *is slope, *A *is selectivity, and [Na^+^] is Na^+ ^concentration. Calibrations were made immediately after withdrawal of the electrodes from the cochlea. Plasma K^+ ^concentrations were obtained using a blood analyzer (Stat Profile M, Nova Biomedical, Waltham, MA).

Data are presented as mean ± sem; n denotes the number of experiments. Differences were considered significant when p < 0.05.

## Results and Discussion

**Figure 1:**
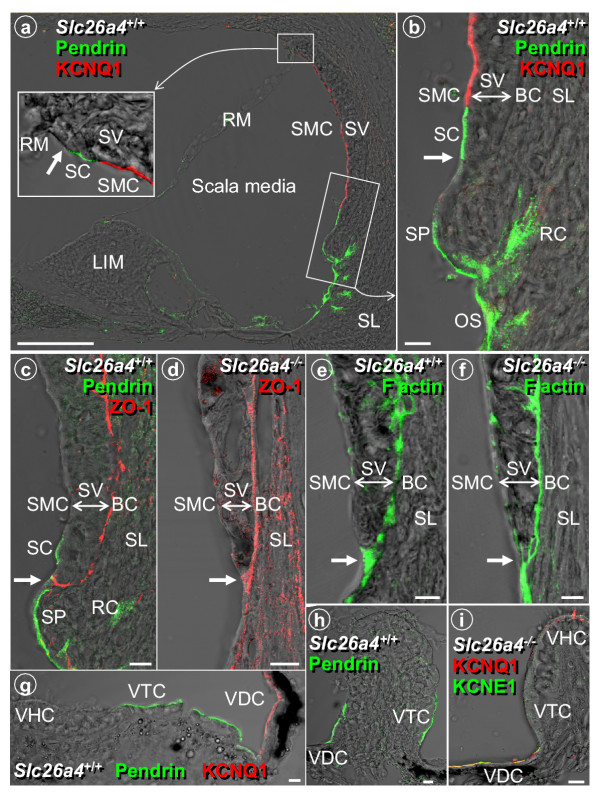
Protein localization of pendrin, KCNQ1, ZO-1 and F actin in cochlea and vestibular labyrinth of *Slc26a4*^+/+ ^and *Slc26a4*^-/- ^mice. **a: **Overview of cochlea; bar = 100 μm. **b-f: **Detail of cochlear lateral wall; bar = 10 μm. **g: **Detail of utricle; bar = 10 μm. **h-i: **Detail of ampulla; bar = 10 μm. RM, Reissner's membrane; SC, spindle-shaped cells, SMC, strial marginal cells; SV, stria vascularis; SL, spiral ligament; LIM, spiral limbus. BC, basal cells; SP, spiral prominence epithelial cells; RC, root cells; OS, outer sulcus epithelial cells; VHC, vestibular hair cells; VTC, vestibular transitional cells; VDC, vestibular dark cells; arrows, basal cells at the top and bottom of stria vascularis form tight junctions with surface epithelial cells.

*In situ *hybridization in the cochlea has suggested that pendrin mRNA is expressed in cells that reside immediately beneath the spiral prominence on the lateral wall of the external sulcus [8]. To determine the location of pendrin protein expression, we performed confocal immunocytochemistry on cryosections prepared from temporal bones of normal (*Slc26a4*^+/+^) mice using an established antibody [3]. Staining was absent when the primary antibody was pre-absorbed with the antigenic peptide (*data not shown*). Strong expression of pendrin was observed not only in outer sulcus epithelial cells, as predicted from *in situ *hybridization data, but also in root cells, in apical membranes of spiral prominence surface epithelial cells and in apical membranes of spindle-shaped cells that are part of stria vascularis (Fig. 1a,1b,1c). The presence of pendrin in spindle-shaped cells suggests that these cells secrete HCO_3 _^- ^into endolymph. Pendrin-mediated HCO_3 _^- ^transport has previously been shown in the kidney [9]. HCO_3 _^- ^in the cochlea may be generated from CO_2 _by carbonic anhydrase located in strial intermediate cells [18,19]. CO_2 _may be supplied by the metabolically highly active stria marginal cells. It is conceivable that pendrin dysfunction interrupts HCO_3 _^- ^secretion and leads to an accumulation of HCO_3 _^- ^in stria vascularis. Preliminary data (Wangemann et al., *unpublished*) support a role for pendrin in HCO_3 _^- ^secretion into endolymph.

The extent of pendrin expression in spiral prominence cells and stria vascularis was determined by labeling KCNQ1, a K^+ ^channel that is expressed in strial marginal cells, and ZO-1, a tight junction protein that labels basal cells and thereby delineates the boundaries of stria vascularis. Dual labeling experiments demonstrated that pendrin is expressed in spindle-shaped cells, which are surface epithelial cells in stria vascularis adjacent to strial marginal cells near the borders of both the spiral prominence and Reissner's membrane (Fig. 1c).

*In situ *hybridization in the vestibular labyrinth suggested that pendrin mRNA is expressed in non-sensory cells [8]. Using confocal immunocytochemistry on cryosections, we observed strong expression of the pendrin protein in the apical membrane of vestibular transitional cells in the utricle and ampullae (Fig. 1g,1h,1i). Dual labeling with KCNQ1 demonstrated that pendrin expression was clearly limited to vestibular transitional cells and did not extend to other non-sensory cells such as vestibular dark cells, which were clearly identified by the expression of KCNQ1 and KCNE1 in their apical membranes.

**Figure 2:**
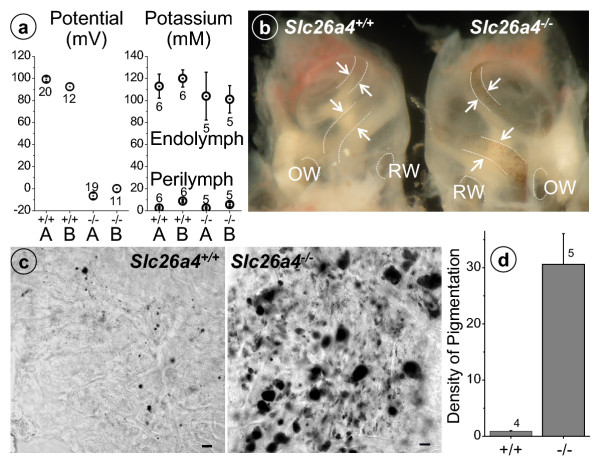
Potential, K^+ ^concentrations and pigmentation of stria vascularis in *Slc26a4*^+/+ ^and *Slc26a4*^-/- ^mice. **a: **Endocochlear potential and K^+ ^concentrations in endolymph and perilymph at the apex (A) and base (B) of the cochlea. Numbers adjacent to symbols denote number of measurements. **b-d: **Pigmentation of stria vascularis in *Slc26a4*^+/+ ^and *Slc26a4*^-/- ^mice. **b: **View of stria vascularis through the bony capsule of the cochlea. OW, oval window, RW, round window; arrows, stria vascularis. **c-d: **Whole-mounts of stria vascularis isolated from age-matched mice. **c: **Laser-scanning images, bar = 10 μm, **d: **Quantification of pigmentation based on optical density.

The onset of pendrin expression during development of the mouse inner ear has been determined to be embryonic day (ED) 13 [14]. Morphologically detectable differences in the inner ears of *Slc26a4*^+/+ ^and *Slc26a4*^-/- ^mice become evident as early as ED 15, when *Slc26a4*^-/- ^mice start to develop an enlarged endolymphatic space that persists into adulthood [14]. Interestingly, sensory hair cells in the cochlea appear normal until postnatal day (PD) 7 but show clear evidence of degeneration by PD 15 [14]. These observations suggest that the cochlear environment supports the survival of sensory hair cells in spite of the enlargement of the endolymphatic duct. A normal endolymphatic K^+ ^concentration, which is critical for hair cell survival [20], is established at PD 3 [21] and may persist in *Slc26a4*^-/- ^at least until PD 7. The time period between PD 7 and 15 is the time when the endocochlear potential develops at the onset of hearing [22]. We hypothesized that a lack of a normal endocochlear potential or an alteration of the endolymphatic K^+ ^concentration could account for deafness in *Slc26a4*^-/- ^mice. Measurements revealed that the endocochlear potential was absent but that the endolymphatic K^+ ^concentration was normal in adult *Slc26a4*^-/- ^(Fig. 2a). No significant differences between *Slc26a4*^+/+ ^and *Slc26a4*^-/- ^mice were found in perilymphatic (Fig. 2a) or plasma K^+ ^concentrations (*Slc26a4*^+/+^, 4.9 ± 0.3 mM, n = 6; *Slc26a4*^-/-^, 5.1 ± 0.3 mM, n = 6). These observations suggest that a primary event leading to deafness in *Slc26a4*^-/- ^mice, and potentially in patients suffering from Pendred syndrome, is the loss of the endocochlear potential. Degeneration of hair cells is probably a response to the loss of the endocochlear potential.

The endocochlear potential is generated by stria vascularis in the lateral wall of the cochlea [17,23]. The potential is generated across the basal cell barrier of stria vascularis by the K^+ ^channel KCNJ10 located in intermediate cells [24], which are connected to basal cells by a high density of gap junctions [25]. Marginal cells of stria vascularis, which form the barrier toward endolymph, transport K^+ ^from the intrastrial space into endolymph and keep the K^+ ^concentration low adjacent to the KCNJ10 K^+ ^channel [26]. To determine the cause of the loss of the endocochlear potential in *Slc26a4*^-/- ^mice, we first determined whether intermediate cells are present in stria vascularis, since a loss of intermediate cells is known to lead to a loss of the endocochlear potential [27,28]. Intermediate cells of stria vascularis were visualized by their pigmentation. Pigmentation was present in stria vascularis of *Slc26a4*^-/- ^mice (Fig. 2b), which suggests that intermediate cells are present. Interestingly, pigmentation of stria vascularis was much stronger in *Slc26a4*^-/- ^than in *Slc26a4*^+/+ ^mice.

**Figure 3:**
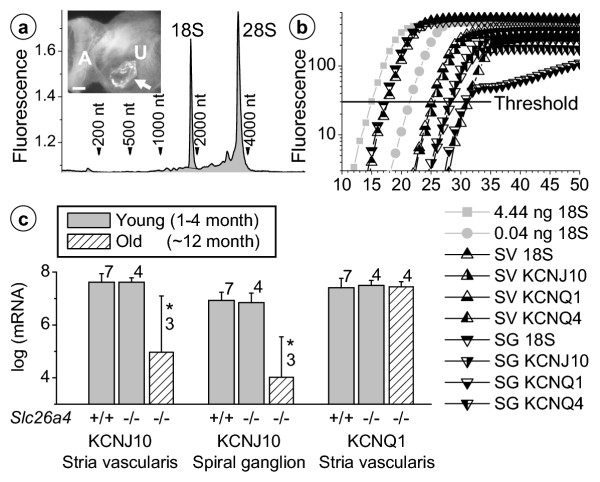
Quantification of KCNJ10 and KCNQ1 mRNA expression in stria vascularis and spiral ganglia of *Slc26a4*^+/+ ^and *Slc26a4*^-/- ^mice. **a: **Electropherogram of total RNA isolated from stria vascularis microdissected from one mouse. The amount of total RNA was obtained from the total integral (*shaded*) and the amount of 18S rRNA was obtained from the integral of the 18S peak. Sharp peaks representing 18S and 28S rRNA demonstrate the high quality of RNA. **Insert: **Genotype of *Slc26a4*^-/- ^mice was verified by the observation of one or few very large rhomboedric otoconia in the utricular macula (*arrow*). A, crista ampullaris; U, utricular macula. Scale bar: 100 μm. **b: **Example of real-time RT-PCR data used for quantification of 18S, KCNJ10, KCNQ1 and KCNQ4. Known quantities of 18S rRNA were used to calibrate the threshold. SV, stria vascularis; SG, spiral ganglia. **c: **Quantification of KCNJ10 and KCNQ1 mRNA in young *Slc26a4*^+/+ ^and young and old *Slc26a4*^-/- ^mice.

To determine in greater detail the cause of the loss of the endocochlear potential in *Slc26a4*^-/- ^mice, we isolated total RNA from stria vascularis and spiral ganglia of young (1–4 month) *Slc26a4*^+/+ ^and both young and old (~12 month) *Slc26a4*^-/- ^mice, assessed amounts of isolated tissues by quantification of 18S rRNA, and quantified the expression of KCNJ10 mRNA. Quantities of stria vascularis isolated from these different mice were similar, since no significant differences were found in the numbers of 18S rRNA molecules (log(rRNA) = 9.46 ± 0.08, n = 17, *pooled data*). In contrast, quantities of spiral ganglia isolated from young and old *Slc26a4*^-/- ^mice (log(rRNA) = 9.04 ± 0.18, n = 4 and 9.29 ± 0.15, n = 6) were significantly smaller than in *Slc26a4*^+/+ ^mice (log(rRNA) = 9.48 ± 0.19, n = 7), consistent with morphometric data (*see below*). Expression of KCNJ10 mRNA was normal in stria vascularis and spiral ganglia of young *Slc26a4*^-/- ^mice but significantly reduced in old *Slc26a4*^-/- ^mice (Fig. 3). Quantifications of KCNQ1 and KCNQ4 mRNA were used to assess possible cross-contamination between the stria vascularis and spiral ganglia preparations based on the assumptions that (1) KCNQ1 is expressed in cells of the stria vascularis but not the spiral ganglia preparation and (2) KCNQ4 is expressed in cells of the spiral ganglia but not the stria vascularis preparation. The number of KCNQ1 mRNA molecules in stria vascularis was 24-fold greater than in spiral ganglia, and the number of KCNQ4 mRNA molecules in spiral ganglia was 5-fold greater than in stria vascularis. These observations validate our measurements of KCNJ10 and KCNQ1 by demonstrating that the microdissected preparations of stria vascularis and spiral ganglia were 98% and 78% pure, respectively.

**Figure 4:**
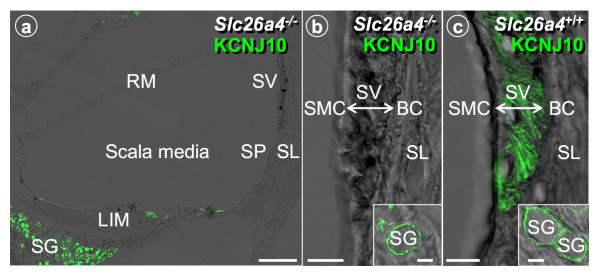
Protein localization of KCNJ10 in the cochlea of *Slc26a4*^+/+ ^and *Slc26a4*^-/- ^mice. **a: **Overview of cochlea; bar = 100 μm. Compare to Fig. 1a to note the enlarged scala media and the distended Reissner's membrane. **b-c: **Detail of lateral wall and spiral ganglia (*insert*); main bar: 10 μm, insert: 5 μm. Expression of KCNJ10 in *Slc26a4*^-/- ^mice was absent in stria vascularis but unchanged in spiral ganglion cells. RM, Reissner's membrane, SV, stria vascularis; SP, spiral prominence; SL, spiral ligament; LIM, spiral limbus; SG, spiral ganglion.

The presence of KCNJ10 mRNA in stria vascularis of *Slc26a4*^-/- ^mice supports the finding that intermediate cells are present. Expression of the KCNJ10 protein was assessed in young *Slc26a4*^-/- ^mice by confocal immunocytochemistry. Interestingly, expression of the KCNJ10 protein was absent in stria vascularis but normal in spiral ganglia (Fig. 4). The absence of the KCNJ10 K^+ ^channel in stria vascularis is sufficient to explain the loss of the endocochlear potential [17].

**Figure 5:**
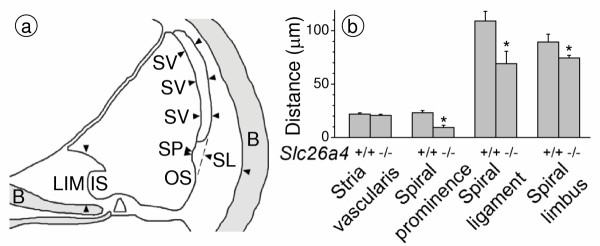
Morphometric analysis of cochlear tissue masses in *Slc26a4*^+/+ ^and *Slc26a4*^-/- ^mice. **a: **locations of measurements. Thickness of stria vascularis (SV) was obtained as average of three distance measurements perpendicular to the surface of marginal cells. Thickness of spiral prominence (SP) was measured perpendicular to a tangential line (*dashed*) that connects the surface of the outer sulcus (OS) with the basal layer of stria vascularis. Thickness of spiral ligament (SL) was measured perpendicular to the tangential line as distance between the surface of spiral prominence and the interface between spiral ligament and bone (B). Thickness of spiral limbus (LIM) was obtained perpendicular to the surface of the bone as a tangential line that touches the inner sulcus (IS) and reaches from the surface of the spiral limbus to the interface between spiral limbus connective tissue and bone. **b: **Summary. Data from 7–8 animals contributed to each column.

**Figure 6:**
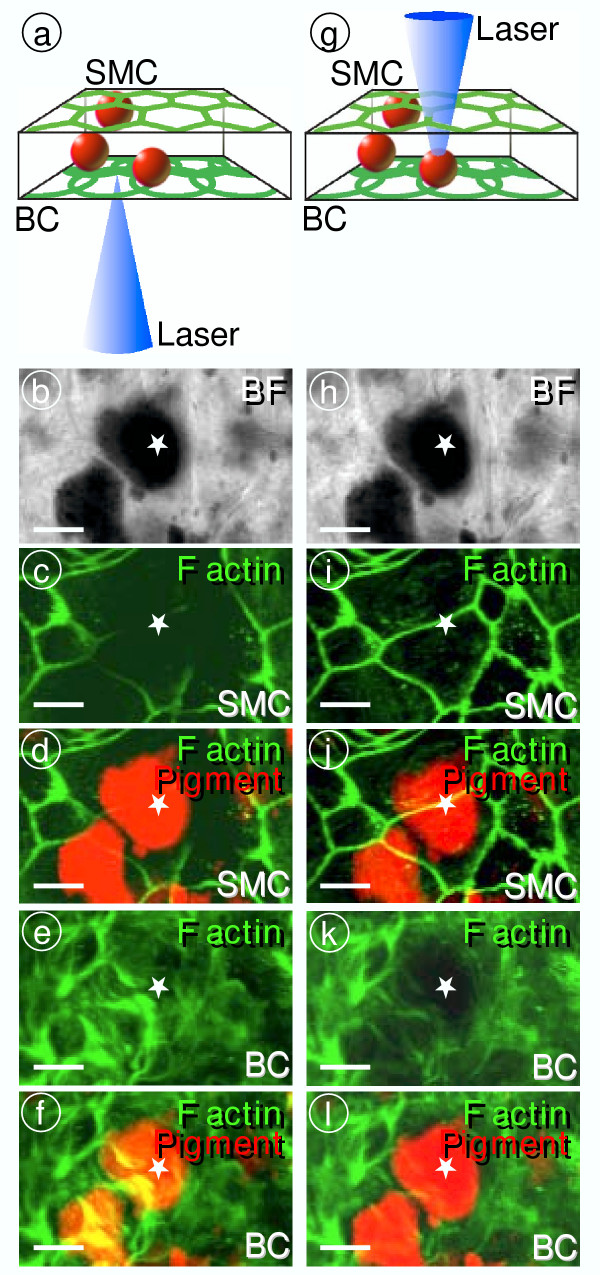
Analysis of marginal and basal cell barriers by in *Slc26a4*^-/- ^mice. Tight junctions were visualized by F actin. Whole-mounts of stria vascularis were viewed either from the basal cell side (**a-f**) or from the marginal cell side (**g-l**). Bright field images verify that the same area was viewed from either side (**b **and **h**). Colored bright field images were mixed with images of F actin staining to indicate the position of pigment granules (**d**, **j**, **f **and **l**). Focus was varied to either visualize the marginal cell barrier (SMC, **c-d **and **i-j**) or the basal cell barrier (BC, **e-f **and **k-l**). Both the marginal cell (**e-f**) and the basal cell barrier (**i-f**) appeared to be intact. It was critical for this finding that pigmentation did not block the path of the laser. Blockage of the laser by pigmentation produces the untrue impression of a discontinuous marginal cell barrier (**c-d**) or basal cell barrier (**k-l**). Comparison of images is aided by marking a significant area with a *star*. Bars = 10 μm.

**Figure 7:**
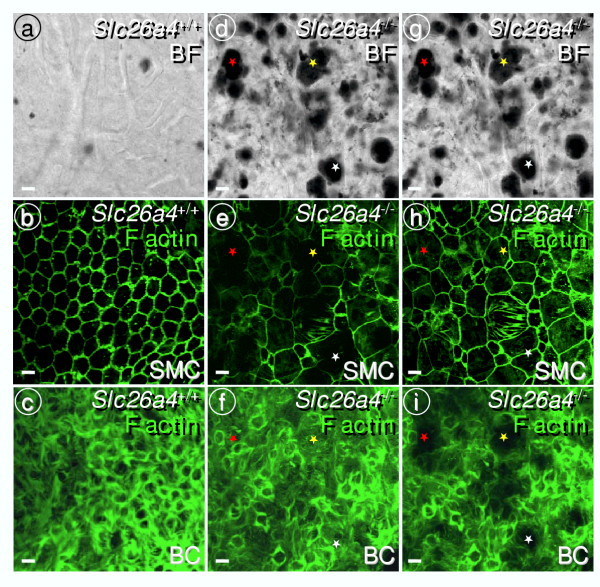
Analysis of marginal and basal cell barriers in *Slc26a4*^+/+ ^and *Slc26a4*^-/- ^mice. Tight junctions were visualized by F actin in whole-mounts of stria vascularis. Bright field images were taken to evaluate pigmentation (**a**, **d **and **g**). Note the intact marginal cell (**b**) and basal cell (**c**) barriers in *Slc26a4*^+/+ ^mice. Minimal pigmentation of *Slc26a4*^+/+ ^mice did not compromise F actin localization. Whole-mounts of stria vascularis from *Slc26a4*^-/- ^mice were viewed either from the basal cell side (**e-f**) or from the marginal cell side (**h-i**). Bright field images verify that the same area was viewed from either side (**d **and **g**). Focus was varied to either visualize the marginal cell barrier (SMC, **b**,**e **and **h**) or the basal cell barrier (BC, **c**,**f **and **i**). Both the basal cell (**f**) and the marginal cell (**h**) barriers appeared to be intact. Blockage of the laser by pigmentation produces the untrue impression of 'holes' in the marginal cell barrier (**e**) or basal cell barrier (**i**). Comparison of images is aided by marking three significant areas with colored *stars*. Bars = 10 μm.

Histological evaluation of cryosections revealed an enlargement of scala media with a large bulging of Reissner's membrane and an apparent degeneration of the organ of Corti, as described earlier [14]. Tissue height of stria vascularis was normal (Fig. 5), consistent with the similar numbers of 18S rRNA molecules in isolated preparations (*see above*). The absence of a change in tissue height is consistent with the presence of intermediate cells [28]. Further, we observed an apparent loss of tissue masses in areas that are normally occupied primarily by type I and II fibrocytes. Spiral prominence in *Slc26a4*^-/- ^mice was less prominent, spiral ligament thinner and spiral limbus flatter (Fig. 5). The observation that tissue masses were lost in the spiral limbus region is consistent with the finding of a reduced number of 18S rRNA molecules in the spiral ganglia preparation (*see above*). In addition, we observed an apparent degeneration of stria vascularis, including an increase in pigmentation and an irregular pattern of the tight junctions of marginal cells (Fig. 2c, 6,7). Tight junctions were visualized by F-actin expression. Marginal cells appeared to form a continuous layer.

Pendrin-expressing surface epithelial cells in the spiral prominence region are located in an area where basal cells, which are interconnected by tight junctions, form additional tight junctions with surface epithelial cells [29]. A discontinuity of this complex junction in *Slc26a4*^-/- ^mice would explain the absence of the endocochlear potential. To evaluate the presence of this connection, we determined by confocal immunocytochemistry the expression of ZO-1 and of F-actin, which associate with tight junction complexes. ZO-1 and F-actin expression revealed a continuous layer of basal cells, including a junction of basal cells with surface epithelial cells in *Slc26a4*^-/- ^mice, as observed in normal mice (Fig. 1c,1d,1e,1f,6,7). These observations make it unlikely that the primary cause of the loss of the endocochlear potential is a compromise in the basal cell barrier.

**Figure 8:**
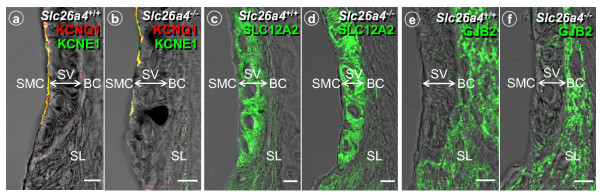
Protein localization of KCNQ1, KCNE1, SLC12A2 and GJB2 in the cochlear lateral wall of *Slc26a4*^+/+ ^and *Slc26a4*^-/- ^mice. **a-f: **bars: 10 μm. SMC, strial marginal cells; SV, stria vascularis; BC, basal cells; SL, spiral ligament.

The observation that endolymphatic and perilymphatic K^+ ^concentrations were normal in *Slc26a4*^-/- ^mice suggests that stria vascularis was able to secrete K^+ ^in spite of the apparent signs of degeneration. The rate of K^+ ^secretion necessary to maintain endolymphatic K^+ ^concentration, however, may be less than necessary in *Slc26a4*^+/+ ^mice given the reduced numbers of sensory hair cells, which provide a major pathway for K^+ ^exit from endolymph [30,31]. In order to substantiate the view that stria vascularis in *Slc26a4*^-/- ^secretes K^+^, we measured the magnitude of the bumetanide-sensitive current exiting stria vascularis across the apical membrane of strial marginal cells and determined the expression of the proteins KCNQ1, KCNE1 and SLC12A2, which are essential for K^+ ^secretion [20,32,33], and of GJB2 (Cx26), which is thought to contribute to K^+ ^cycling [23]. K^+ ^secretion is known to be sensitive to 10^-5 ^M bumetanide, a loop-diuretic that inhibits the Na^+^/2Cl^-^/K^+ ^cotransporter SLC12A2 [26]. Bumetanide-sensitive currents were found in both *Slc26a4*^+/+ ^and *Slc26a4*^-/- ^mice although the magnitude of the current was significantly smaller in *Slc26a4*^-/- ^mice (22 ± 6 μA/cm^2^, n = 4 versus 14 ± 2 μA/cm^2^, n = 4). KCNQ1 and KCNE1, subunits of the secretory K^+ ^channel, were co-localized in the apical membrane of strial marginal cells; the Na^+^/2Cl^-^/K^+ ^cotransporter SLC12A2, which is located in the basolateral membrane of strial marginal cells, was found in stria vascularis; and the gap junction protein GJB2 was found in spiral ligament of *Slc26a4*^+/+ ^and *Slc26a4*^-/- ^mice (Fig. 8). Co-localization of KCNQ1 and KCNE1 proteins was also observed in vestibular dark cells (Fig. 1i). Expression of KCNQ1 protein in stria vascularis of *Slc26a4*^-/- ^mice is consistent with the finding of KCNQ1 mRNA expression (Fig. 3). These observations make it unlikely that the primary cause of the loss of the endocochlear potential is a compromise of K^+ ^secretion by strial marginal cells or a compromise of gap junction mediated K^+ ^cycling.

## Conclusions

**Figure 9:**
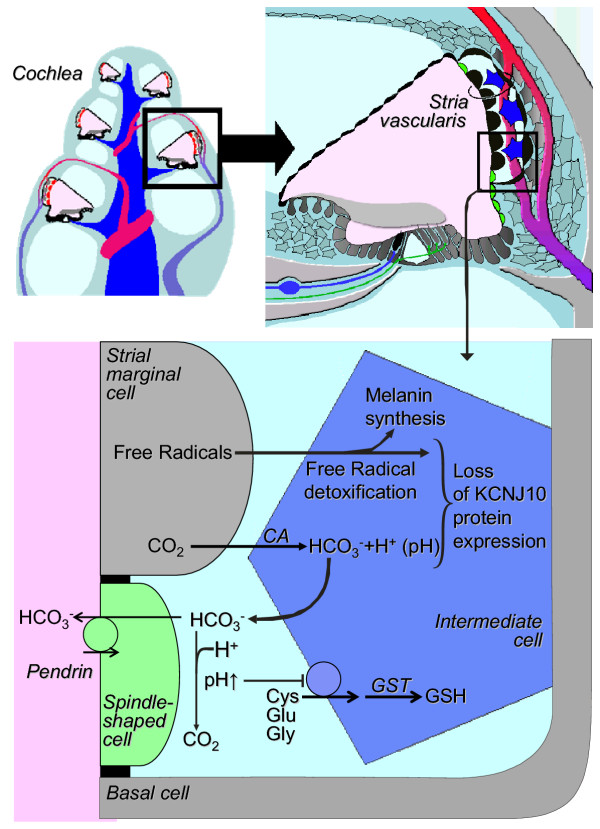
Model for the loss of KCNJ10 in the absence of pendrin expression in stria vascularis. Cys, cysteine, Glu, glutamate, Gly, glycine, CA, carbonic anhydrase, GST, glutathione-S-transferase, GSH, glutathione.

We described locations of pendrin expression in the cochlea and vestibular labyrinth and detected normal endolymphatic K^+ ^concentrations in spite of an enlargement of this fluid compartment. We found that *Slc26a4*^-/- ^mice lack the endocochlear potential because they do not express KCNJ10 protein. Intermediate cells protect stria vascularis by converting CO_2 _to HCO_3 _^- ^and by detoxifying free radicals (Fig. 9). CO_2 _and free radicals are generated by the large numbers of mitochondria in the metabolically highly active strial marginal cells. Intermediate cells employ carbonic anhydrase to convert CO_2 _to HCO_3 _^- ^[18,19] and catalase to detoxify free radicals. To protect themselves from free radical damage, intermediate cells generate glutathione and melanin pigment [34-37]. It is conceivable that loss of pendrin, which may secrete HCO_3 _^- ^into endolymph, results in an accumulation of HCO_3 _^- ^and an alkalinization of the intrastrial spaces. This extracellular alkalinization may enhance free radical stress, since it may inhibit the uptake of cysteine and thereby limit production of the protective glutathione [38]. Support for the hypothesis of enhanced free radical stress comes from the observed hyperpigmentation in mice lacking pendrin. Strial hyperpigmentation has also been observed in other conditions that are associated with free radical stress, such as acoustic trauma [37]. Alterations in the cytosolic pH in conjunction with free radical stress may lead to the loss of KCNJ10 protein expression in strial intermediate cells. Function and expression of other K^+ ^channels has been shown to be controlled by the cytosolic pH and free radicals, which encode the metabolic state of the cell [39]. Suppression of the KCNJ10 K^+ ^channel in strial intermediate cells, which is essential for the generation of the endocochlear potential, is probably the direct cause of deafness in *Slc26a4*^-/- ^mice and patients suffering from Pendred syndrome.

## Competing interests

None declared.

## Authors' contributions

PW designed and coordinated the immunocytochemical, morphometrical and molecular biological experiments. EMI and BA carried out confocal immunocytochemistry on cryosections. SJ and SVJ carried out confocal microscopy on whole-mounts of stria vascularis. SVJ and RJM carried out quantitative RT-PCR. DCM designed and coordinated electrophysiological experiments. TW carried out measurements of the endocochlear potential and the endolymphatic K^+ ^concentration. JHL carried out current measurements on strial marginal cells. SMW, LAE, IER and EDG provided the mice and the pendrin-specific antibody. PW and DCM conceived the study. PW wrote the manuscript. All authors read and approved the final manuscript.

## Pre-publication history

The pre-publication history for this paper can be accessed here:


